# Prescribing antipsychotics in child and adolescent psychiatry: guideline adherence

**DOI:** 10.1007/s00787-020-01488-6

**Published:** 2020-02-12

**Authors:** Mariken Dinnissen, Andrea Dietrich, Judith H. van der Molen, Anne M. Verhallen, Ynske Buiteveld, Suzanne Jongejan, Pieter W. Troost, Jan K. Buitelaar, Pieter J. Hoekstra, Barbara J. van den Hoofdakker

**Affiliations:** 1 Department of Child- and Adolescent Psychiatry, University Medical Center Groningen, University Center of Child and Adolescent Psychiatry, University of Groningen, P.O. Box 660, 9700 AR Groningen, The Netherlands; 2 Department of Clinical Psychology and Experimental Psychopathology, University of Groningen, Groningen, The Netherlands; 3 Department of Biomedical Sciences of Cells and Systems, Section of Cognitive Neurosciences, University Medical Center Groningen, University of Groningen, Groningen, The Netherlands; 4 De Bascule Child- and Adolescent Psychiatry Center Amsterdam, Amsterdam, The Netherlands; 5 Department of Cognitive Neuroscience, Donders Institute for Brain, Cognition and Behaviour, Radboud University Nijmegen Medical Center, Nijmegen, The Netherlands; 6 Karakter Child and Adolescent Psychiatry University Center, Nijmegen, The Netherlands

**Keywords:** Antipsychotics, Prescription, Children, Adolescents, Guidelines

## Abstract

Antipsychotics are often prescribed to children and adolescents, mostly off-label. We aimed to assess adherence to recommendations of guidelines for antipsychotic prescription. We reviewed 436 medical records from 155 clinicians from 26 clinics within three Dutch child and adolescent psychiatry organizations (*n* = 398 outpatient, *n* = 38 inpatient care). We assessed target symptoms, diagnostic process, prior and concomitant treatment, and consideration of contra-indications. Multiple logistic regression assessed the role of age, sex, and psychiatric diagnosis on adherence to three main recommendations: to (1) prescribe antipsychotics only after other treatments proved insufficient, (2) always combine antipsychotics with psychosocial interventions, and (3) not prescribe multiple antipsychotics simultaneously. Most patients received off-label antipsychotics. Main target symptoms were inattention/hyperactivity (25%), aggression (24%), and other disruptive behaviors (41%). Most patients underwent diagnostic evaluation before the first prescription; however, screening of contra-indications was low (0.2–19%). About 84% had previously received psychosocial treatment and 48% other psychoactive medication, but 9% had not received any treatment. Notably, only 37% continuously received concomitant psychosocial treatment. Simultaneous use of multiple antipsychotics occurred in 3.2%. Younger children were at higher risk of non-adherence to guideline recommendations regarding prior and concomitant treatment, children with autism spectrum disorder or attention-deficit/hyperactivity disorder more likely not to receive concomitant psychosocial treatment. Sex did not significantly affect adherence. Our findings implicate insufficient adherence to important recommendations regarding antipsychotic use in children and adolescents. Especially younger children are at higher risk of receiving suboptimal care. There is an urgency to consistently offer psychosocial interventions during antipsychotic treatment.

**Electronic supplementary material:**

The online version of this article (10.1007/s00787-020-01488-6) contains supplementary material, which is available to authorized users.

## Introduction

The prescription of antipsychotics in children and adolescents is mostly off-label and it is largely unknown how well prescribing clinicians adhere to available clinical guidelines concerning these agents. Antipsychotics are mostly prescribed for target symptoms such as disruptive behaviors, anxiety, irritability, and sleep problems [1–4], and often for longer periods of time than recommended [5, 6]. This off-label use of antipsychotics may be hazardous, as the effects and possible adverse effects have not yet been studied adequately [7]. Adverse effect profiles are not the same for each antipsychotic agent [8] but can include weight gain, development of diabetes, sedation, extrapyramidal symptoms, increased prolactin levels, and an increased QT-interval [9–12]. Children are more vulnerable than adults to develop these often harmful side effects, which makes adherence to existing guidelines in this age group particularly essential [13].

There is a multitude of guidelines that address prescribing antipsychotics in children and adolescents, e.g. disorder-specific guidelines, such as for the treatment of psychoses or schizophrenia [14], autism spectrum disorders (ASD) [15], bipolar disorder [16], or behavioral disorders [17–19], or guidelines addressing the use of antipsychotics in general [20–24]. Several studies have evaluated monitoring of antipsychotic use in clinical practice [25–30], but studies on the initiation of antipsychotics are scarce.

To our knowledge, only two such studies have been conducted. The first study involved a survey amongst prescribers of Medicaid-insured children who had received a prescription for an antipsychotic [7]. Their survey assessed whether the prescription met an FDA approved indication and whether it was in accordance with best practice adherence as per the American Academy of Child and Adolescent Psychiatry’s (AACAP) “Practice Parameter for the Use of Atypical Antipsychotic Medication in Children and Adolescents” [22]. Results indicated that in about half of the cases clinicians indicated they were compliant with the evaluated recommendations regarding appropriate target symptoms, prior treatment, age, dosing, and not combining multiple antipsychotics in prescribing antipsychotics [7]. Results from this study may be biased as data was obtained from the prescribers, which may have caused clinicians to answer in a socially desirable way. Moreover, not all best practice recommendations were addressed due to practical constraints. The second study also focused on Medicaid-insured children and evaluated recommendations that were based on a review of guidelines from the United States [31], addressing antipsychotic polypharmacy, dosing, prior treatment, metabolic screening, and monitoring. The authors found substantial shortcomings across recommendations [32]. However, they noted that not all relevant data might be reflected in the claims database that was used to obtain information on the course of treatment. Furthermore, generalizability of the results of both studies may be limited, as they only included Medicaid-insured children, resulting in a sample from mostly lower-income families.

For the current study, our objective was to obtain a more objective assessment of adherence to guidelines regarding the initiation of antipsychotic prescription in children and adolescents by clinicians from clinics for child and adolescent psychiatry in children who had been prescribed an antipsychotic. We, therefore, reviewed information from medical records instead of asking the prescribing clinician. We also looked into guideline adherence regarding changes in treatment that were relevant to the antipsychotic prescription, such as discontinuation of concomitant treatment or the addition of a second antipsychotic. Furthermore, we aimed to identify whether children’s age, sex, and primary clinical diagnosis would be associated with guideline adherence. We focused on children without intellectual disabilities, as adherence to prescription guidelines in children and adolescents with intellectual disabilities had been topic of a previous paper from our group [33].

## Methods

### Sample

We aimed to randomly select 500 medical records, equally distributed across three large mental health organizations for general child and adolescent psychiatry that provide both inpatient and outpatient treatment in different parts of the Netherlands. These organizations provide care for a broad spectrum of patients with all kinds of mental health problems (ranging from mild to severe), including youngsters with different ages, and socioeconomic, educational, and cultural backgrounds; and each organization consisted of several outpatient clinics in different locations. We selected records from randomized lists of patients who had received a prescription for an antipsychotic or had an appointment for possible psychopharmacological treatment in 2012. Subsequently, we screened each record and reviewed if it met our inclusion criteria. Records were included if the patient in question: (1) had received a prescription for an antipsychotic agent in the year 2012; (2) had their first antipsychotic prescription at the same organization the record was reviewed; (3) was < 18 years old at the time of the first prescription; and (4) had an intelligence quotient of 70 or higher or, if this was unknown, attended regular education at the time of the first prescription. While patients were selected from those having had an antipsychotic prescription in 2012, initial start, which was assessed in this study could be in 2012 or before.

### Measures

In the Netherlands as in Europe in general, there is no single, comprehensive guideline on prescribing antipsychotics in children and adolescents. To determine on which points to assess guideline adherence, we identified those recommendations that were shared by most available guidelines, resulting in 8 recommendations (Table 1). We reviewed several national as well as international guidelines, practice parameters, and recommendations focusing on autism spectrum disorders, disruptive behavior, psychosis, tics, or more general on off-label use of medication or antipsychotics. These were only considered if they were published before 2013 in either English or Dutch. Furthermore, we considered quality concepts formulated by Kealey et al. [31] relating to the initiation of antipsychotic treatment.

**Table 1:** Recommendations on antipsychotic prescription in children and adolescents that are shared by most Dutch as well as international guidelines

*Diagnostics*
1. A thorough diagnostic assessment should be conducted prior to the antipsychotic prescription
*Prior treatment (psychosocial and/or pharmacological)*
2. Before the initiation of an antipsychotic agent is warranted, other recommended psychosocial or psychopharmacological treatment options should be attempted and be found insufficient
*Initiation of antipsychotic treatment*
3. Antipsychotics should be aimed at specific target symptoms
4. Patients should be screened for contra-indications in the patient or family history, such as the presence of diabetes, hyperlipidemia, or cardiovascular disease^a^
5. Parents and children should be educated about the antipsychotic, for example on possible side effects, the off-label status of the medication, the importance of lifestyle adjustments, and the need to regularly attempt discontinuation
6. Dosing should start low
*Concomitant treatment*
7. Antipsychotics should always be prescribed in combination with psychosocial interventions
8. Multiple antipsychotics should not be prescribed simultaneously

Full information on guidelines that were used, as well as excerpts from these guidelines resulting in these recommendations, can be accessed in the electronic supplementary materials

^a^In line with most guidelines, we focused on the assessment of contra-indications in the patient or family history, rather than of laboratory measures

To assess adherence to these 8 recommendations, we examined each medical record using a comprehensive checklist that covered adherence to the recommendations listed in Table 1, as well as a number of patient characteristics. All information on assessment and prior treatment was based on the situation prior to the very first antipsychotic prescription, concomitant treatment was reviewed during up to 3 years of antipsychotic treatment or until the antipsychotic treatment was discontinued. We reviewed both paper and electronic records, when available. Some more recent electronic systems had a template for recording length, weight, and blood pressure.

### Procedure

Data were collected between January 2016 and June 2017 by five research assistants, who all had a master-degree in either psychology or a related field, had been extensively trained in the screening and reviewing procedures, and had regular meetings to reach consensus.

After completion of the paper checklist, data was entered in an electronic database. About 12% of the paper checklists (*n* = 52) was entered twice to check for the error rate in the data-entering process.

### Data analysis

We assessed frequencies and percentages to categorical checklist items that were relevant for assessing adherence to the 8 recommendations. Additionally, we aimed to assess predictors for three of these, representing the most important recommendations. These main recommendations were selected post hoc, based on the completeness of the information that could be derived from the medical record and that were of most interest. We selected recommendation 2, 7, and 8 (Table 1) regarding (1) prescribing antipsychotics only after other treatments proved insufficient, (2) always combining antipsychotics with psychosocial interventions, and (3) not prescribing multiple antipsychotics simultaneously. Patients who were diagnosed with a psychotic disorder (*n* = 4) were scored as adhering to recommendations 2 and 7, as for this indication, first-line antipsychotic treatment and antipsychotic monotherapy is on-label. In Europe antipsychotics are not licensed for the treatment of irritability in autism spectrum disorders and was thus considered as off-label. We used multiple logistic regression to assess the predictive value of age and sex on adherence vs non-adherence to these three main recommendations as the outcome variable. We also included the effects of primary psychiatric diagnosis on guideline adherence. For this, we categorized groups of children who, according to their record, were diagnosed with an ASD (irrespective of comorbidity), attention-deficit/hyperactivity disorder (ADHD; irrespective of comorbidity but without ASD), or behavioral disorder (without ADHD or ASD), according to the hierarchy of diagnoses used in the DSM-IV (Diagnostic and Statistical Manual of Mental Disorders, fourth edition). Additionally, we defined a group of other DSM-IV diagnoses and a group without any psychiatric diagnoses, but because of large heterogeneity in the former group and a low sample size in the latter, we excluded these groups from the logistic regressions. Finally, we included all possible interactions between age, sex, and primary psychiatric diagnosis into the regression. Covariates with *p* < 0.2 were considered in multivariate analyses; backwards selection eliminated variables with *p* < 0.1. Multicollinearity was assessed using variance inflation factors (VIF). Patients for whom guideline adherence with respect to one of the three main recommendations was unknown were excluded from these analyses. To check whether this affected results, we performed sensitivity analyses combining the group of unknown adherence with the non-adherence group.

## Results

### Patient characteristics

Although we aimed to review equal amounts of records from each organization, in one of them, only 52 met the inclusion criteria. The other two organizations had larger numbers of medication visits in 2012. Therefore, we reviewed additional records in these organizations to achieve 192 in each, resulting in a total sample of 436. Due to time and financial constraints, we did not achieve our goal of 500 records.

Patient characteristics are described in Table 2. Boys (76.1%) had a mean age of 9.55 (SD 3.07) years old at the time of their first prescription and were significantly younger than girls, whose mean age was 12.0 (SD 3.84) years, *t* (147) = − 5.98, *p* < 0.001. Detailed information on frequencies of psychiatric diagnoses in our sample is given in appendix II. Notably, only 4 (0.9%) of our patients were diagnosed with a psychotic disorder at the time of their first antipsychotic prescription. None of our patients were diagnosed with bipolar disorder.

**Table 2:** Patient characteristics at the time of the first antipsychotic prescription: age, sex, intellectual functioning, primary psychiatric diagnosis, and treatment setting

	Total sample (*n* = 436)
Age, mean (SD) [range], y	10.1 (3.43) [3.33–17.9]
Sex, *n* (%)	
Male	332 (76.1%)
Female	104 (23.9%)
Intellectual functioning, *n* (%)	
Borderline (TIQ 70–79)	60 (13.8%)
Low average (TIQ 80–89)	92 (21.1%)
Average (TIQ 90–109)	126 (28.9%)
High average (TIQ 110–119)	39 (8.9%)
Superior (TIQ 120–129)	27 (6.2%)
Very superior (TIQ > 129)	5 (1.1%)
Not reported	87 (20.0%)
Primary DSM-IV axis I diagnosis, *n* (%)	
Autism spectrum disorder	229 (52.5%)
Attention deficit/hyperactivity disorder	82 (18.8%)
Disruptive behavior disorder	21 (4.8%)
Other DSM-IV diagnoses^a^	61 (14.0%)
No diagnosis	43 (9.9%)
Treatment setting in which antipsychotic was prescribed, *n* (%)	
Inpatient	38 (8.7%)
Outpatient	398 (91.3%)

*DSM-IV* Diagnostic and Statistical Manual of Mental Disorders, fourth edition; TIQ, total intelligence quotient

^a^This category includes all other axis I diagnoses mentioned in the DSM-IV

### Diagnostics

Before receiving their first antipsychotic prescription, most patients (89.9%) had undergone a complete diagnostic evaluation, whereas for others prescription had been done while the assessment was ongoing (5.3%) or the prescription was already given during the first consult (1.8%). For the remaining 3%, this information could not be derived from the medical records. Several informants were used during the diagnostic assessment. In 96.8% of the records, diagnostic activities were reported with the child, such as observation (37.8%), neuropsychological testing (25.5%), questionnaires (19.5%), a semi-structured interview (2.3%), and/or an unspecified meeting with a clinician (72.7%). In 96.6% were diagnostic activities reported with one or both parents, including use of questionnaires (55.3%), semi-structured interviews (3.9%), and/or an unspecified interview with a clinician (89.9%). Furthermore, developmental histories were obtained in 83%, somatic histories in 58%, and a psychiatric family history in 59.4%. Finally, in 72.2% of the patients, was information obtained from another informant, such as school or day care (70.4%), a grandparent (0.7%), or a prior clinician (3.4%).

### Prior treatment

Patients had received a large variety of psychosocial treatments before their first antipsychotic prescription. The majority (83.7%) had previously received some form of psychosocial treatment, and about half (48.4%) had received one or more other psychopharmacological agents; predominantly stimulant medication (Table 3). In 37 patients (8.5%) was the antipsychotic prescription not preceded by any other (psychosocial or psychopharmacological) treatment.

**Table 3:** Psychosocial and psychopharmacological treatment patients initiated before their first antipsychotic prescription

	Total sample (*n* = 436)
*Prior psychosocial treatment, n (%)*	
No prior psychosocial treatment reported	71 (16.3%)
CBT parents (manualized parent management training)	29 (6.7%)
Non-CBT parent contacts (nonspecific/eclectic parent support or counseling)	268 (61.5%)
Psycho-education	49 (11.2%)
School interventions (nonspecific advice, support)	9 (2.1%)
Individual or group CBT	33 (7.6%)
Non-CBT individual or group treatment	162 (37.2%)
Day treatment	100 (22.9%)
Inpatient treatment or living arrangements away from parents	85 (19.5%)
Other unspecified psychosocial treatment	7 (1.61%)
*Prior psychopharmacological treatment, n (%)*	
No prior pharmacological treatment reported	225 (51.6%)
Stimulant	186 (42.7%)
Alpha-adrenergic agent	21 (4.8%)
Anxiolytic	15 (3.4%)
Antidepressant	13 (3.0%)
Non-stimulant ADHD medication	3 (0.7%)

*CBT* cognitive behavioral therapy

### Initiation of the antipsychotic treatment

Table 4 shows the year of the first antipsychotic prescription, the profession of the prescribing clinicians, the type of antipsychotic that was initially prescribed with average start doses and the target symptoms that were mentioned in the record as the reason(s) for which the antipsychotic was prescribed. In 11.9% were no target symptoms reported. The start dose did not exceed the maximum recommended dosage in any of the patients. Table 4 further shows the risk factors based on the patient and family history that were assessed prior to the first prescription as well as the patient education that was provided to patients and their parents.

**Table 4:** Initiation of the antipsychotic: year of prescription, clinician background, type, and dosage of antipsychotic and target symptoms

	Total sample (*n* = 436)	
*Year of first antipsychotic prescription, n (%)*		
1998	1 (0.2%)	
1999	1 (0.2%)	
2000	1 (0.2%)	
2002	3 (0.7%)	
2003	6 (1.4%)	
2004	5 (1.1%)	
2005	7 (1.6%)	
2006	13 (3.0%)	
2007	19 (4.4%)	
2008	27 (6.2%)	
2009	45 (10.3%)	
2010	46 (10.6%)	
2011	104 (23.9%)	
2012	158 (36.2%)	
*Prescribing clinician, n (%)*		
Child and adolescent psychiatrist	310 (71.1%)	
Other physician	97 (22.2%)	
Nurse (practitioner)	4 (0.9%)	
Pediatrician	1 (0.2%)	
Prescriber not reported	24 (5.5%)	
*Type of antipsychotic, n (%)*		*Start dose, mean (SD), mg*^*a*^
Risperidone	298 (68.3%)	0.4 (0.3)
Pipamperone	46 (10.6%)	12.0 (8.9)
Aripiprazole	40 (9.2%)	2.7 (1.4)
Olanzapine	29 (6.7%)	2.9 (1.5)
Haloperidol	9 (2.1%)	0.9 (1.2)
Quetiapine	7 (1.6%)	35.5 (34.8)
Pimozide	2 (0.5%)	0.5 (0.0)
Trial with multiple types of antipsychotics	2 (0.5%)	
Type of antipsychotic not reported	3 (0.7%)	
*Target symptoms, n (%)*		
Symptoms of attention deficit/hyperactivity disorder	109 (25.0%)	
Aggression	106 (24.3%)	
Anxiety	80 (18.3%)	
Sleep problems	56 (12.8%)	
Problems with sensory processing	45 (10.3%)	
Irritability	30 (6.9%)	
Disruptive behaviors other than aggression and irritability^b^	180 (41.3%)	
Tics	30 (6.9%)	
Rigidity	26 (6.0%)	
Psychotic symptoms	24 (5.5%)	
Other target symptoms	21 (4.8%)	
Depressive symptoms	16 (3.7%)	
Emotion regulation	15 (3.4%)	
Eating problems	12 (2.8%)	
No target symptoms reported	52 (11.9%)	
*Risk factors verbally screened prior to first antipsychotic prescription, n (%)*		
History of cardiovascular problems	32 (7.3%)	
Family history of cardiovascular problems	83 (19.0%)	
History of obesity	14 (3.2%)	
Family history of obesity	5 (1.1%)	
History of diabetes	4 (0.9%)	
Family history of diabetes	14 (3.4%)	
History of dyslipidemia	1 (0.2%)	
Family history of dyslipidemia	2 (0.5%)	
History of hypertension	8 (1.8%)	
Family history of hypertension	8 (1.8%)	
*Type of information provided, n (%)*		
Potential side effects	127 (29.1%)	
Potential beneficial effects	81 (18.6%)	
Off-label status antipsychotic	25 (5.7%)	
Possibility of requiring blood draw	9 (2.1%)	
Importance of short term use and discontinuation	7 (1.6%)	
Importance of healthy diet	5 (1.1%)	
Importance of exercise	4 (0.9%)	
Possibility of not yet reported side effects	0 (0%)	
Information was given, but content was not specified	67 (15.4%)	
No information reported	258 (59.2%)	

^a^Percentage in which the start dose was unknown: Risperidone: 6.4%; Pipamperone: 10.9%; Aripiprazole: 2.5%; Olanzapine: 6.9%; Haloperidol: 0%; Quetiapin: 14.3%; Pimozide: 0%

^b^This includes oppositional behaviors or behavioral problems, anger, and temper tantrums other than aggression and irritability

A total of 155 different clinicians from 26 clinics within our participating organizations for child and adolescent psychiatry gave the first antipsychotic prescriptions in our sample. The average number of patients per clinician was 2.76 (SD 2.67, range 1–18), which did not differ between organizations (*F*(2,155) = 0.23, *p* = 0.79).

### Concomitant treatment

We found that in the 3 years after the initial prescription, only 37.4% of the patients received antipsychotic medication that was continuously accompanied by psychosocial treatment as recommended. In 20% this information could not be derived from the medical record. It further appeared difficult to obtain data on the exact time periods of non-concomitant treatment, however, we were certain that 42.7% of the patients had a period of at least 1 month during which the antipsychotic was not combined with any psychosocial treatment.

Most patients only received one type of antipsychotic at the same time (93.8%), but 3.2% had a more extended period during which they received multiple types simultaneously, other than a transitional period of switching from one antipsychotic to another. For the other 3%, this information could not be derived from the medical records.

### Predictors of adherence to main guideline recommendations

Results of multiple logistic regression analyses on the three main recommendations (1) try other treatment options first; (2) always combine with psychosocial treatment; (3) avoid multiple antipsychotics) are summarized in Table 5. Age was significantly associated with adherence to recommendation (1) and (2), i.e., younger children were more likely not to have had any prior pharmacological or psychosocial treatment and to receive an antipsychotic without concomitant psychosocial treatment at some point during treatment. Furthermore, the primary clinical diagnosis was significantly associated with adherence to recommendation (2): i.e., children with a primary diagnosis of ASD or ADHD were more likely not to receive concomitant psychosocial treatment; however, effect sizes were small. Sex, although having a significant effect on adherence to recommendation ii in the univariate regression, was not a significant factor in the multivariate regression. Finally, adherence to recommendation iii was not significantly influenced by sex, age, or primary diagnosis. Sensitivity analyses for each recommendation including unknown adherence showed similar results. There was no evidence of multicollinearity (VIFs < 1.03).

**Table 5:** Univariate and multivariate logistic regression for predictors of non-adherence to main recommendations

	Univariate		Multivariate	
	*B* (SE *B*)	OR (95% CI)	*B* (SE *B*)	OR (95% CI)
*Recommendation 1: try other treatment options first*^ab^				
Sex^c^	− 1.28 (0.62)*	0.28 (0.08–0.93)		
Age	− 0.24 (0.70)***	0.78 (0.68–0.90)	− 0.24 (0.70)***	0.78 (0.68–0.90)
Diagnosis				
ASD (reference)				
ADHD	− 0.62 (0.56)	0.54 (0.18–1.62)		
DBD	NA^d^	NA^d^		
*Recommendation 2: always combine with psychosocial treatment*^ab^				
Sex^c^	− 0.94 (0.26)***	0.39 (0.24–0.65)		
Age	− 0.21 (0.04)***	0.81 (0.76–0.87)	− 0.13 (0.04)**	0.88 (0.81–0.95)
Diagnosis				
ASD (reference)				
ADHD	− 0.31 (0.31)	0.77 (0.43–1.40)	− 0.30 (0.31)	0.74 (0.40–1.34)
DBD	− 1.39 (0.51)**	0.25 (0.09–0.68)	− 1.47 (0.52)**	0.23 (0.08–0.64)
*Recommendation 3: avoid multiple antipsychotics*^a^				
Sex^c^	− 0.57 (0.57)	0.57 (0.19–1.73)		
Age	0.02 (0.08)	1.02 (0.87–1.20)		
Diagnosis				
ASD (reference)				
ADHD	1.10 (1.07)	3.00 (0.37–24.34)		
DBD	− 1.02 (0.83)	0.36 (0.07–1.82)		

*ADHD* attention deficit/hyperactivity disorder, *ASD* autism spectrum disorder, *DBD* disruptive behavior disorder, *NA* not available

*p < .05. **p < .01. ***p < 0.001

^a^0 = adhered, 1 = not adhered

^b^Patients who were diagnosed with a psychotic disorder (*n* = 4) were scored as adhering to these recommendations, as for this indication, first-line antipsychotic treatment and antipsychotic monotherapy is on-label

^c^0 = male, 1 = female

^d^00% adherence

### Agreement rate

For the 52 checklists that were entered twice to check for the error rate in the data-entering process, a total amount of 41 errors were detected across the 752 numeric variables that were included in the assessment of agreement in the data-entering process. This resulted in an error rate of 0.1% and, therefore, an agreement rate of 99.9%, which we considered sufficient.

## Discussion

In this medical record review, we found mixed adherence to recommendations regarding antipsychotic prescribing in children and adolescents. The recommendation that antipsychotics should continuously be prescribed with concomitant psychosocial treatment was especially disregarded; in over half of the patients this was not always the case. Fortunately, most antipsychotic treatments were only initiated after the use of other treatment options and patients rarely received multiple antipsychotics simultaneously. Younger children were more at risk of receiving an antipsychotic without any prior treatment and to not receive concomitant psychosocial treatment at some point during treatment. EMA-approved indications for antipsychotic treatment (i.e. aggression in conduct disorder combined with subaverage intelligence, psychotic symptoms or severe manic episodes in bipolar-I disorder in adolescents [34–37]) were present in only 5.5% of our sample, 12% of the records contained no mention of any target symptoms.

Sufficient prior treatment was also found by Rettew et al. [7], who reported that in only 1.2% of the cases, patients did not receive any prior psychosocial treatment and in 5.4% of the cases, there was no prior pharmacological treatment. Other similarities to their study are the low incidence of prior treatment with cognitive behavioral interventions and the fact that younger children were less likely to have received prior treatment. In contrast to our findings, in over half of a sample of Medicaid insured children, there was no prior psychosocial treatment in the 3 months before the antipsychotic had been prescribed [32, 38]. However, our study, as well as the study by Rettew et al. [7] assessed all prior lifetime treatments, which might explain this difference. Additionally, international or regional differences in attitudes towards psychotropic drug use, insurance coverage, or availability of psychosocial treatment options may explain variability between studies [39, 40]. Of note, no patients in our study received antipsychotics for mania as part of a bipolar disorder. This may be due to a lower administrative prevalence of childhood-onset bipolar disorder in Europe compared to the US [41].

A great concern is that only 37% of the children and adolescents continuously received concomitant psychosocial interventions. Periods of treatment with only an antipsychotic occurred more in younger children and patients with ASD and with ADHD. In other studies, sole antipsychotic use varied from 33% [42] to 60% [43], whereas Rettew et al. [7] reported that the majority of their sample also received other interventions, such as psychotherapy or interventions at school. This can be explained by the fact that we evaluated concomitant treatment over a longer period of time (a maximum of three years after the first prescription) and Rettew et al. only inquired about concomitant treatment at the time the survey was completed.

We found that in only 3% of the patients in our sample multiple antipsychotics had been combined. In contrast, another study in the United States [44] found that in hospitalized children, this percentage was even 14%. Especially violent or aggressive children and those previously hospitalized were at higher risk, but also children with intellectual disabilities, psychotic disorders, and developmental disorders. The exclusive focus on an inpatient population in the latter study may explain the higher percentage. This suggests that the usage of multiple antipsychotics in outpatient settings may be of lesser concern. Additionally, not including patients with intellectual disabilities is an important difference from previous studies, which may explain dissimilar results.

We also examined a number of guideline recommendations that have not been evaluated previously, because they could not be extracted from large databases or were too specific to be asked in relatively short surveys. For example, we found that most patients had undergone a complete diagnostic evaluation prior to their first prescription, which is important because only after proper diagnostics can treatments be tailored to the patient. Less positive were our findings regarding consideration of contra-indications, with limited screening of medical risk factors in the patient and family history. Furthermore, better education of the patient and the parents about antipsychotics, their effects, and lifestyle changes may be needed, as guidelines stress the importance of proper patient education [22, 23]. Clinicians may, however, have performed these steps, but not recorded them in the medical record. As mentioned, although some more recent electronic patient records contained templates for monitoring physical parameters, other essential steps leading up to prescribing antipsychotics were not included.

Our study has several strengths when compared to other studies in the field. It was the first to use medical records to comprehensively assess guideline adherence in prescribing antipsychotics to children and adolescents without intellectual disabilities. This provided a more objective assessment compared to, large patient databases on care utilization or clinician surveys. Furthermore, we included a large sample including both inpatient and outpatient children and adolescents from a large number of prescribing clinicians spread across the country, representing a cross-section from different organizations for child and adolescent psychiatry in the Netherlands.

However, our study also has some shortcomings. Most importantly, our exclusive reliance on medical records also had drawbacks. Especially in older paper records information was often limited, possibly leading to an overestimation of adherence problems, particularly in patients who had received their first prescription longer before our sample year 2012. While treatment history from previous treatment settings was mostly well reported in the medical record of the organizations where this study was conducted, we cannot rule out that some details may not have been included. Nevertheless, the most crucial information on our three main recommendations was generally well documented, thus not hampering our main conclusions. Another limitation is that we only considered a period of 1 month to assess adherence to guideline recommendations with regard to required concomitant psychosocial treatment, which may have been overly stringent with families may have started psychosocial later due to vacations or illness on the part of either the patient or therapist; consequently, this relatively short time period may suggest an overestimation of non-adherence. Furthermore, we unfortunately did not have data on the number of patients lost to follow up during the first 3 years of antipsychotic treatment, making longitudinal outcomes harder to interpret. Finally, from our data it was not possible to identify a broader range of potential predictors of adherence. Previous studies demonstrated that lower social economic status, higher problem severity, poor parenting style, and clinician factors such as profession or more years of experience negatively influence guideline adherence associated with the prescription of antipsychotics [30, 44]. Future studies may further investigate the role of clinician, patient or contextual characteristics on guideline adherence. Finally, the extent to which our results can be generalized to international populations is unclear. On the one hand, our sample was comparable to samples from previous studies regarding children’s’ sex and diagnoses [7, 33]—and our findings were generally in line with previous studies from other countries. However, we cannot exclude differences in treatment and diagnostic culture, insurance, and availability of treatment options between or within countries to have an impact on guideline adherence [39, 40]. Future large-scale studies may shed more light on regional differences. Additionally, studies comparing those patients who did receive an antipsychotic to those in which this might have been considered could shed more light on predictors of guideline adherence regarding the choice of treatment. Also of influence might be the combination of psychiatric diagnosis and the specific target symptoms for which the antipsychotic is considered. This could also be of interest for future studies.

We speculate that the lack of one unambiguous and comprehensive European (or Dutch) prescription guideline for antipsychotic treatment in children and adolescents may have negatively influenced prescription practice in our sample. In our view, efforts should be put into developing a uniform, international guideline, incorporating recent developments in research on antipsychotic treatment in children and adolescents.

## Conclusion

We assessed guideline adherence in the initiation of antipsychotic use in children and adolescents without intellectual disabilities by retrospectively reviewing medical records from out- and inpatient child and adolescent mental health care organizations in the Netherlands. Our findings raise concern about insufficient adherence to a number of important guidelines in clinical practice. Younger children were even more at risk of receiving suboptimal care, warranting special attention to this vulnerable group. The high prevalence of antipsychotic prescriptions that was not continuously combined with psychosocial interventions is particularly worrisome. Important points of improvement would include a more standardized documentation of the essential treatment steps in the medical record, better education of clinicians, and development of a uniform, comprehensive and widely accessible guideline on prescribing antipsychotics in children and adolescents.

## Electronic supplementary material

Below is the link to the electronic supplementary material.

Supplementary material 1 (DOCX 27 kb)

## Abbreviations

ADHD: Attention-deficit/hyperactivity disorder
ASD: Autism spectrum disorder
DSM-IV: Diagnostic and Statistical Manual of Mental Disorders, fourth edition
