# Supplementary material for: Prescribing antipsychotics in child and adolescent psychiatry: guideline adherence
Source: Eur Child Adolesc Psychiatry. 2020 Feb 12;29(12):1717–27. doi: 10.1007/s00787-020-01488-6 (PMC7641940; doi:10.1007/s00787-020-01488-6)
Supplement: Supplementary file 1 — Supplementary material 1 (DOCX 27 kb) [file 787_2020_1488_MOESM1_ESM.docx]

**Prescribing antipsychotics in child and adolescent psychiatry: guideline adherence**

**Electronic supplementary material**

# Table 1. Guidelines on the prescription of antipsychotics in children and adolescents that served as the basis for constructing the most important recommendations as well as a checklist to assess guideline adherence in this study.

| **Abbreviated Name** | **Sponsoring Organization and Guideline Title** | **Year** | **Country** |
| --- | --- | --- | --- |
| AACAP-AAA (American Academy of Child and Adolescent Psychiatry 2011) | AACAP—Practice parameter for the use of atypical antipsychotic medications in children and adolescents | 2011 | USA |
| AACAP-PsyMed (American Academy of Child and Adolescent Psychiatry 2009) | AACAP—Practice parameter on the use of psychotropic medication in children and adolescents | 2009 | USA |
| AACAP-BP (McClellan *et al* 2007) | AACAP—Practice parameter for the assessment and treatment of children and adolescents with bipolar disorder | 2007 | USA |
| AACAP-ODD (Steiner *et al* 2007) | AACAP—Practice parameter for the assessment and treatment of children and adolescents with oppositional defiant disorder | 2007 | USA |
| AACAP-SZ (American Academy of Child and Adolescent Psychiatry 2001) | AACAP—Practice parameter for the assessment and treatment of children and adolescents with schizophrenia | 2001 | USA |
| TRAAY (Pappadopulos *et al* 2003) | Center for the Advancement of Children’s Mental Health—Treatment recommendations for the use of antipsychotics for aggressive youth | 2003 | USA |
| T-MAY (Scotto Rosato *et al* 2012) | Center for Education and Research on Mental Health Therapeutics—Treatment of maladaptive aggression in youth | 2012 | USA |
| SGA-GP (Horn *et al* 2012) | Prescribing second-generation antipsychotic medications: Practice guidelines for general practitioners | 2012 | CA |
| ACCARE-META (Kalverdijk, L.J., Helfrich, E. 2010) | Accare Center for child and adolescent psychiatry—[translated] Monitoring for metabolic and endocrine side effects of antipsychotics | 2010 | NL |
| NVvP-ASD (Nederlandse Vereniging voor Psychiatrie 2009) | Dutch Association for psychiatry (NVvP)— [translated] Guideline for the diagnosis and treatment of autism spectrum disorders in children and youths | 2009 | NL |
| NVvP-OFF (Nederlandse Vereniging voor Psychiatrie 2012) | Dutch Association for psychiatry (NVvP)— [translated] Guidance for prescribing off-label medication in child and adolescent psychiatry | 2012 | NL |

# Abbreviations: AACAP, American Academy of Child and Adolescent Psychiatry; CA, Canada; NL, the Netherlands; USA, United States of America.

# Table 2. Recommendations on antipsychotic prescription in children and adolescents that are shared by most Dutch as well as international guidelines^a^

| **DIAGNOSTICS** | | |
| --- | --- | --- |
| 1. A thorough diagnostic assessment should be conducted prior to the antipsychotic prescription. | | |
|  | AACAP-AAA | “(…) careful diagnostic assessment, attention to comorbid medical conditions (…)” |
|  | TMAY | “Conduct a thorough initial evaluation and diagnostic work-up before initiating treatment.” |
|  | SGA-GP | “Ensure the appropriate psychiatric diagnosis is made” |
| **PRIOR TREATMENT (Psychosocial and/or pharmacological)** | | |
| 2. Before the initiation of an antipsychotic agent is warranted, other recommended psychosocial or psychopharmacological treatment options should be attempted and be found insufficient. | | |
|  | AACAP-AAA | “For some conditions, AAAs serve as a primary treatment (e.g., schizophrenia). In other circumstances, AAAs are generally only used after other interventions, both psychosocial and pharmacological, have failed (e.g., disruptive behavior disorders).” |
|  |  | “In the absence of specific FDA indications or substantial empirical support for the use of AAAs for other specific problems (e.g., disruptive behavior disorders) in populations of children and adolescents, clinicians should consider other pharmacological or psychosocial treatment modalities with more established efficacy and safety profiles prior to the onset of AAA use.” |
|  | AACAP-ODD | “Medications may be helpful as adjuncts to treatment packages, for symptomatic treatment and to treat comorbid conditions” |
|  | AACAP-BP | “Psychotherapeutic interventions are an important component of a comprehensive treatment plan for early-onset bipolar disorder” |
|  | TRAAY | “Begin with psychosocial and educational treatment.” |
|  | TMAY | “Recommendations 10 and 11 pertain to psychosocial interventions, which should be the first line of treatment because of its lower risk, preceding the use of medication to address aggression except in emergency circumstances…” |
| **INITIATION OF ANTIPSYCHOTIC TREATMENT** | | |
| 3. Antipsychotics should be aimed at specific target symptoms. | | |
|  | AACAP-ODD | “Medications may be helpful as adjuncts to treatment packages, for symptomatic treatment and to treat comorbid conditions. Typical and atypical antipsychotics are helpful in treating aggression after appropriate psychosocial interventions have been applied in the context of mental retardation and pervasive developmental disorders.” |
|  | SGA-GP | “Primary care physicians should identify specific target symptoms, approved indications, and the degree of functional impairment before initiating treatment with a second-generation antipsychotic” |
|  | NVvP-ASD | [translated] “It is recommended to primarily treat the symptoms that are the primary cause of dysfunction.” |
|  | NVvP-OFF | [translated] “Record the target symptom in the medical chart.” |
| 4. Patients should be screened for contra-indications in the patient or family history, such as the presence of diabetes, hyperlipidemia, or cardiovascular disease | | |
|  | AACAP-AAA | “Due to the specific risks associated with the use of AAAs, additional factors to address, prior to the initiation of treatment with the AAAs, include obtaining a personal and family history of diabetes and hyperlipidemia, seizures and cardiac abnormalities, as well as any family history of previous response or adverse events associated with AAAs.” |
|  | AACAP-BP | “Psychopharmacological interventions require baseline and follow-up symptom, side effect, and laboratory monitoring as indicated….The American Diabetes Association’s recommendations for managing weight gain for patients taking antipsychotics should be followed. This includes baseline BMI, waist circumference, blood pressure, fasting glucose, and a fasting lipid panel.” |
|  | AACAP-SZ | “The use of antipsychotic agents requires….. documentation any required baseline and follow-up laboratory monitoring...” |
|  | SGA-GP | “The family history should include questions about diabetes (type 1, type 2, gestational), hyperlipidemia, cardiovascular disease, schizophrenia, schizoaffective disorder, psychosis not otherwise specified, and bipolar disorder. The personal history should include questions about smoking, physical activity, screen time (e.g., computers, TV), and use of sugar-sweetened beverages.” |
| 5. Parents and children should be educated about the antipsychotic, for example on possible side effects, the off-label status of the medication, the importance of lifestyle adjustments, and the need to regularly attempt discontinuation | | |
|  | AACAP-AAA | “(…) thorough discussion of the risks and benefits of psychotropic treatment with both the youth and their guardians.” |
|  | TMAY | “Educate about side effects” |
|  | SGA-GP | “Physicians should inform patients taking second-generation antipsychotics of the need for preventive lifestyle practices (e.g., healthy eating, physical activity).” |
|  | ACCARE-META | [translated] “Educate on risk of weight gain and the importance of prevention, healthy diet, (…), sufficient exercise, signals of hyperprolactinemia (…) discuss duration of use (3 months, 1 year) and discuss need for blood draw in due time.” |
|  | NVvP-OFF | [translated] “Inform parents on the off-label use and record their informed consent in the medical chart. The prescribing psychiatrist is obligated to inform parents or the patient on the off-label treatment (advantages, disadvantages, risks and known side effects (...).” |
| 6. Dosing should start low. | | |
|  | AACAP-AAA | “Dosing of the AAAs should follow the “start low and go slow” approach and seek to find the lowest effective dose, recognizing that dosing may differ based on the targeted symptoms and patient diagnosis …(lacking other evidence) the upper limit of dosage of an AAA should not exceed the maximum recommended dose prescribed for adults” |
|  | AACAP-SZ | “The use of antipsychotic agents requires…long-term monitoring to reassess dosage needs, dependent on the stage of illness. Higher doses may be required during the acute phases, with smaller dosages during the residual phases.” |
|  |  | “The medication dosage should be periodically reassessed to ensure that the lowest effective dose is being used” |
|  | TRAAY | “Physicians should use a conservative dosing strategy (“start low, go slow”)” |
| **CONCOMITANT TREATMENT** | | |
| 7. Antipsychotics should always be prescribed in combination with psychosocial interventions. | | |
|  | AACAP-AAA | “Prior to the initiation of and during treatment with an AAA, the general guidelines that pertain to the prescription of psychotropic medications should be followed… including education and psychotherapeutic interventions for the treatment and monitoring of improvement” |
|  | AACAP-ODD | “Medication should not be the sole intervention in ODD” |
|  | AACAP-BP | “Psychotherapeutic interventions are an important component of a comprehensive treatment plan for early-onset bipolar disorder” |
|  | AACAP-SZ | “Adequate treatment requires the combination of psychopharmacological agents plus psychosocial interventions” |
|  | T-MAY | “Provide or assist the family in obtaining evidence-based parent and child skills training during all phases of care” |
|  |  | “Treatment planning should consider a multimodal approach. Education of the parents and child and forming a team of healthcare professionals is just as important in this phase of treatment as it is during previous ones. Their input will help you to better understand the potential for unexpected risks and benefits and may result in more appropriate monitoring of patients.” |
|  | TRAAY | “Psychosocial and educational interventions should continue after medication treatment begins.” |
|  | NVvP-OFF | [translated] “Consider non-pharmacological interventions at any moment” |
| 8. Multiple antipsychotics should not be prescribed simultaneously. | | |
|  | AACAP-AAA | “The simultaneous use of multiple concurrent AAAs has not been studied rigorously and generally should be avoided.” |
|  | AACAP-PsyMed | “….there is limited evidence in children and adolescents for the use of two antidepressants or two antipsychotics as an initial treatment approach or as a specific endpoint for treatment. However, it is not uncommon for patients to be taking two antidepressants or two antipsychotics at the same time when transitioning for one medication to another.” |
|  | T-MAY | “Use of two simultaneous psychotropic medications should be avoided.” |

Abbreviations: AAA, atypical antipsychotic; BMI, body mass index; FDA, United States Food and Drug Administration; ODD, oppositional defiant disorder.

^a^ Full guideline information (full name, organization, year, country, reference) is given in table 1.

# Table 3. Frequencies of axis I diagnoses in this study

| **DSM-IV axis I diagnoses**, n (%) | **Total sample** (n=436) |
| --- | --- |
| Learning disorders | 13 (3.0%) |
| Motor skills disorders | 5 (1.1%) |
| Communication disorders | 12 (2.8%) |
| Pervasive developmental disorders (including autism spectrum disorders) | 229 (52.5%) |
| Attention deficit/hyperactivity disorder | 153 (35.1%) |
| Disruptive behavior disorder | 67 (15.4%) |
| Tic disorder | 33 (7.6%) |
| Elimination disorders | 19 (4.4%) |
| Disorder of infancy, childhood, or adolescence NOS | 6 (1.4%) |
| Substance-related disorders | 2 (0.5%) |
| Psychotic disorder | 4 (0.9%) |
| Mood disorder NOS | 5 (1.1%) |
| Depressive disorders | 19 (4.4%) |
| Anxiety disorders | 30 (6.9%) |
| Attachment disorders | 16 (3.7%) |
| Gender identity disorders | 2 (0.5%) |
| Eating disorders | 23 (5.3%) |
| Sleep disorders | 2 (0.5%) |
| Impulse-control disorders | 3 (0.7%) |
| Adjustment disorders | 8 (1.8%) |

Abbreviations: NOS, not otherwise specified
